# Understanding of Diabetes in Tibetan, Mongolian, Miao, Dai, Uygur, and Yi Medicine and Collation of Prevention and Cure Medicines

**DOI:** 10.1155/2022/9308598

**Published:** 2022-03-30

**Authors:** Yi Shi, Banghua Zhou, Liqiong Yu, Yaqin Liu, Yunfeng Han, Xi Tang, Xianrong Lai

**Affiliations:** ^1^School of Pharmacy, Chengdu University of Traditional Chinese Medicine, Chengdu 611137, China; ^2^Qiandongnan Engineering and Technology Research Center for Comprehensive Utilization of Ethnic Medicine, Kaili University, Kaili 556011, China

## Abstract

Diabetes seriously endangers human health and causes a huge economic burden. With the improvement of people's living standard, the prevalence of diabetes is getting higher and higher, and age is becoming younger. It is an increasingly serious global problem. Therefore, it is imperative to find the drugs to treat diabetes. Ethnic medicine is an important part of the world's medicinal treasure house and has its own unique system. This study systematically combined the theoretical understanding of the prevention and treatment of diabetes of Tibetan, Mongolian, Miao, Dai, Uygur, and Yi people by searching the existing literature studies published until 2021, library collection resources (related ethnic monographs, medical books, standards of medicinal materials, etc.), CNKI, PubMed, and other databases and collected and sorted the relevant medicines. A total of 112 kinds of ethnic medicines for the prevention and treatment of diabetes have been discovered, including plant medicines (105 kinds), animal medicines (6 kinds), and fungal medicines (1 kind). The composition of family and genus, medicinal parts, and life forms of medicinal plants were analyzed, and the number of drugs used in the prevention and treatment of diabetes in each ethnic group was statistically analyzed. The results showed that Rosaceae was at the top of the list, and the drugs used in underground parts accounted for 33.90% of the total, and the medicinal plants were mainly herbaceous, and the Mongolians have the largest number of diabetes medicines. In addition, CNKI, PubMed, and other databases selected “medicinal materials name,” “diabetes,” and “hypoglycemia” as keywords, the top 30 medicinal materials reported in existing literature were listed, and their Chinese name, the Latin name of the original plant, family and genus, nationality used, medicinal parts, and active ingredients related to the prevention and treatment of diabetes were introduced in detail. Among the 30 medicines, Astragalus membranaceus, Pueraria lobata, and Coptis chinensis are the most commonly used. Through literature research, this study summarized the existing theories of ethnic medicine for the prevention and treatment of diabetes, collected and sorted out ethnic medicine, clarified the potential mechanism of ethnic medicine, and provided effective data compilation. Ethnic medicine has a long history of treating diabetes, and there are abundant medicinal materials, to provide a new idea and basis for treating diabetes.

## 1. Introduction

Diabetes is a lifelong disease characterized by hyperglycemia distinguished by insufficient insulin secretion and/or dysfunction, resulting in metabolic disorders such as carbohydrates, fats, and proteins. Currently, about 463 million adults (aged 20–79) worldwide have diabetes, and the number is expected to reach 700 million by 2045. In 2019, 10% of global health spending went on diabetes treatment, and 79% of adults with diabetes live in low- and middle-income countries [1]. The number of patients with diabetes is high, the cost of treatment is very high, and the long-term acceptance of Western medicine is prone to drug resistance, which will cause some damage to the liver and kidney function of patients [2]. In particular, in the later stage of disease progression, diabetic retinopathy, diabetic nephropathy, diabetic neuropathy, diabetic foot, and other complications caused by diabetes lead to poor quality of life of patients. Therefore, seeking effective prevention and treatment methods has always been a hot topic in medical research.

China's Tibetan, Mongolian, Miao, Dai, Uygur, Yi, and other ethnic medicines have a long history and unique knowledge in the prevention and treatment of diabetes. Ethnic medicine has rich theoretical and practical experience in the treatment of diabetes [3, 4], which can effectively delay the disease. Moreover, the resources of ethnic medicine for treating diabetes and its complications are abundant, with abundant practical experience and multicomponent, multitarget, high coordination, low price, and multiple organ functions. It has incomparable advantages over Western medicine. However, most of the existing literature studies are about the medical system of Tibetan or single ethnic group, but there are no systematic arrangement and discussion on the theoretical understanding of diabetes and treatment drugs in the medical classics of ethnic minorities. Therefore, this study sorted out and elaborated the treatment of diabetes by ethnic medicine, summarized the theoretical understanding, condensed the previous prevention and treatment experience, and expected to provide a new way of thinking and promote the research progress of expanding the treatment of diabetes.

## 2. Materials and Methods

This study adopts the research method of literature mining, takes “diabetes” and “hypoglycemia” as keywords, starts with the medical and pharmaceutical classics and literature studies of Tibetan, Dai, Mongolian, Miao, Uygur, and Yi ethnic minorities, local drug standards, CNKI, PubMed, and other databases, and manually searches the existing literature studies published until 2021, library collection resources, etc. This article comprehensively collected and sorted Tibetan, Dai, Mongolian, Miao, Uygur and Yi medical literature related to diabetes prevention and treatment (e.g., Gan Lu Si Bu, Mongolian Medicine canon, The Four Medical Tantras, Jing Zhu Materia Medica, Dai Pharmacy, Dang Ha Ya Long, Baidi Medical Book, Mongolian Pharmacy, Eight essence and local medicinal materials standards, etc.). By combing and summarizing historical literature, we can theoretically understand the existing research, diagnosis, and treatment ideas and characteristics of diabetes etiology and pathogenesis, and explore related drugs for diabetes prevention and treatment. According to the Chinese name, the Latin name of the original plant, the name of the family to which it belongs, the nationality used, the medicinal part, the active ingredient, and so on summarize its rules and characteristics.

## 3. Results

### 3.1. Understanding of Diabetes among Different Nationalities

#### 3.1.1. Understanding of Diabetes in Tibetan Medicine

Tibetan medicine is an important part of Chinese traditional medicine. It is a traditional medicine created and developed by the Tibetan people in their long medical practice. It is mainly distributed in the five Tibetan areas of Tibet, Qinghai, Sichuan, Gansu, and Yunnan. With “three causes and five sources” as its theoretical core, Tibetan medicine constitutes a unique, scientific, and complete Tibetan medicine system. “Three causes” refer to lung, Chiba, and bacon to maintain the normal function of the human body; “Five sources” refer to wind, air, fire, water, and earth, which constitute all things in the world. Tibetan doctors believe that under healthy conditions, a dynamic balance is maintained between the three factors and seven essences (diet, blood, meat, fat, bone, marrow, and semen) which constitute the material basis of the human body, between the organs and tissues of the human body, and between the human body and the external environment. Once the balance is broken, it will lead to disease [5].

Tibetan medicinal knowledge of diabetes, which first appeared in the eighth century, was born in *the Four Medical Tantras* (the classics of Tibetan medicine). The book calls it “jingnisaku disease,” which falls under the category of frequent urination and is caused by a combination of internal and external factors such as uncontrolled diet and irregular daily routines [6, 7]. According to color and nature of urine, the Tibetan medical book *Eight Branches Essentials* puts forward the classification of diabetes and elaborates the diseases that diabetic patients are prone to, which coincides with the idea of various acute and chronic complications in the late stage of diabetes recognized by modern medicine [8, 9]. Tibetan medicine believes that diabetes is a cold disease. Taking warm and hot medicines is the main treatment, supplemented by living therapy (away from cold and humid areas, increasing activity), diet therapy (avoid eating sweet, salty, cold food), and external treatment [6,10].

#### 3.1.2. Understanding of Diabetes in Mongolian Medicine

Mongolian medicine is gradually accumulated by the Mongolian people by their traditional medical practice experience, absorbing the essence of Tibetan, Chinese, and ancient Indian medical theory, combined with the characteristics of their nomadic nationality. Mongolian medicine is mainly based on the theory of “three roots,” which also includes the theory of yin-yang and five elements, five elements, seven elements, and six basic symptoms. Three of these are Khii (power of various physiological functions), Xir (heat), and Batgan (a mucous nutrient in the human body, characterized by cold). The seven elements are the essence of diet (nutrient solution), blood, muscle, fat, bone, bone marrow, and semen and the essence that makes the basic substance of human body constantly biochemical. According to Mongolian medicine, the three roots of human body are the three kinds of energy and basic substances that maintain human life activities and physiological functions and play an energy supply and dominant role in the physiological activities of the seven elements [11].

Mongolian medicine refers to diabetes as “xiherixijing disease [12],” “thirst elimination disease [13],” or “urine drip disease [14].” Due to the influence of Tibetan medicine, Mongolian medicine has a similar understanding of diabetes to Tibetan medicine. They believe that it is caused by excessive consumption of sweet, salty, greasy, and nondigestible food, irregular sleep and rest, resulting in an imbalance between the three roots and seven elements of human body, resulting in Batgan and fat overhang, and fighting with Khill and Xir, which can lead to the liver and kidney hot and decline of function and cannot transform the essence needed by the human body. Thus, it is mixed with the abnormal decomposition of dross into the urine and excreted out of the body, resulting in dizziness, headache, dry mouth, thirst, slight heat, lower abdominal pain, more drinking and eating, becoming thinner, frequent urination, visual impairment, and other symptoms of thirst-quenching disease [15, 16]. Mongolian medicinal treatment of diabetes is based on the starting point of disease cause and the overall theory and mainly uses turbidity removal liquid of Batgan to promote the normal metabolism of the essence and dregs in the body, maintain the balance between the three roots and seven elements, and combine with the lesion site for the treatment according to symptoms [17, 18].

#### 3.1.3. Understanding of Diabetes in Miao Medicine

Miao medicine is originated and developed from the production and living practice of the Miao people. Miao doctor emphasized that “all diseases arise from poison, and poison is the source of all diseases.” “Poison theory” is the theoretical basis of etiology and pathology and the gist of Miao doctor's diagnosis and treatment of diseases and guidance of clinical practice [19,20]. Miao doctors believe that toxicity, deficiency, injury, accumulation, bacteria, and insects are the six factors causing human diseases, but all of them can cause disease in the way of toxicity [21].

Miao doctors call diabetes “gangnuo” (urine-sweet syndrome), and its causes are related to diet, congenital factors, old age, weakness, environment and climate, etc. [22]. Eating out of control and overwork lead to dysfunction of the viscera, which is “internal poison.” Cold, heat, wind, frost, rain, dew, fog, and other environmental and climatic conditions are also regarded as pathogenic factors, which can lead to wind poison, gas poison, water poison, cold poison, fire poison, wet poison, phlegm poison, and other invasions of the human body and disease, which is “external poison.” The interaction between “external poison” and “internal poison” lead to the accumulation of “poison” in the body, which cannot be transformed and excreted out of the human body, resulting in the occurrence of diabetes. Miao doctors mainly diagnose diabetes by observing the urine of patients. If the urine attracts ants to eat and suck, it can be identified as gangnuo, so it is also called ant syndrome. For the treatment of gangnuo, Miao doctors mainly adopt the first principle of “detoxification as the method” and mainly use the methods of clearing poison (a method to neutralize toxins in the body by using drugs with opposite properties according to the different properties of toxins and pathogens), removing poison (a method for detoxifying with strong and powerful drugs), and attacking poison (a method of using toxic drugs to attack the virulence) [23, 24].

#### 3.1.4. Understanding of Diabetes in Dai Medicine

Dai medicine refers to a complete set of medical theory systems with distinctive ethnic that Dai people have been fighting against diseases for a long time, taking their own medical ideology and culture as the main body and constantly absorbing and referring to part of Chinese medicine and ancient Indian medicine. It takes palm leaf culture as the background, “four pagodas” and “five Skandhas” as the theoretical core, “three plates” theory as the physiological and pathological basis, and “jieya” theory as the diagnosis and treatment characteristics [25]. The four pagodas are the four elements of earth, fire, water, and wind. Dai doctors believe that human beings are inseparable from nature. Earth, fire, water, and wind are the basic elements of all things in nature, as well as the four basic substances of human body. The five Skandhas are the free translation of the Sanskrit Skandha, namely color, perception, perception, thought, and action [26]. They are aggregates of various material and spiritual elements of the human body.

From the perspective of Dai medicine, long-time metabolic abnormalities occur in patients with endocrine and metabolic diseases such as diabetes, and metabolic abnormalities are mostly attributed to toxins in the body. Therefore, the abnormal metabolism in the early stage of diabetes is due to the “sugar poison” in the body. The essence of diabetes is caused by the imbalance of four pagodas and five Skandhas and is inseparable from the pagoda and fire pagoda. The earth pagoda includes more than 20 species of the viscera, liver, lung, spleen, kidney, large intestine, hair, teeth, fingers, toes, bones, and muscles. Under normal circumstances, the earth pagoda maintains the physiological activities of human organs, such as the physiological functions of the five Zang organs and the growth and development of skeletal muscles. Once the earth pagoda is out of order, it will lead to human health problems. For example, if the earth pagoda is insufficient, there will be symptoms such as lack of food, palpitation, muscle wasting, weakness of muscles and bones, dysuria of urine, and urine. If the earth pagoda is sufficient, there will be some symptoms such as general or local stiffness and cold, loss of warmth, nausea, and vomiting [27]. The fire pagoda represents the Yang Qi in the human body, including the role of fire in the growth and development of the human body. For example, only fire can be digested and absorbed after eating, and sufficient fire can make the human body healthy and disease-free. If the fire pagoda is insufficient, there will be dizziness, palpitation, cold waist and limb syncope, abdominal pain, diarrhea, indigestion, and other symptoms; excessive fire pagoda will also lead to human fever, dry mouth, thirst, sweating, sore throat and gums, sore tongue, dry stool, and other symptoms [28,29]. Therefore, in view of the “sugar poison” occurring in the pre-diabetes period, Dai medicine first adopted the “jieya” rule, aiming at relieving toxins in the body with medicines and maintaining the balance and coordination between the four pagodas. Then, by determining the type and degree of the four pagoda lesion of diabetic patients, the clinical Dai medical college was set up to adjust the four pagodas to treat the disease.

#### 3.1.5. Understanding of Diabetes in Uygur Medicine

Uighur medical theory is a relatively complete ethnic pharmacy with its own characteristics, which is gradually accumulated through long and difficult medical practice on the basis of widely absorbing relevant theories such as Chinese medicine, ancient Indian medicine, Arab medicine, and Persian and ancient Greek medicine. Uygur medicine takes the theory of “four substances” and the theory of “temperament” as the theoretical core [30]. The four substances refer to fire (sun, fire in nature), gas (air, wind), soil, and water in nature, which is similar to the “four pagodas” theory of Dai medicine. The four major substances are the material basis of life activities, and the birth, growth, prosperity, and decline of all things in the world are influenced and acted upon by them [11]. According to the Uygur medicine, the four major substances in nature are under the influence of the four “temperaments” of the human body and take various nutrients as raw materials and produce four kinds of “heliti” (translated as body fluid in Chinese) through the normal function of the liver, namely bile substance, blood substance, mucinous substance, and black bile substance. The four kinds of heliti are constantly consumed and replenished in a dynamic balance to maintain the normal operation of the body and ensure the health of the human body. According to the Uygur medicine theory, diabetes is divided into two kinds: heat and cold. It is the imbalance of the body under the abnormal influence of four substances. When the bile substance changes too much, it causes pancreatic secretion dysfunction, abnormal glucose metabolism, and blood sugar rise, reduces renal function and glomerular filtration rate, and then excretes sugar with urine, so that heat diabetes is formed; when the kidney qi balance suffers destruction because of cold invasion and lost the ability of absorption, digestion, and excretion, the organization inside kidney produces change, so that cold diabetes is formed [31, 32]. To reduce blood sugar, eliminate symptoms, improve immunity, and prevent complications, Uygur doctors often adopt systematic treatment methods such as diet therapy, exercise therapy, and drug therapy to treat diabetes [33].

#### 3.1.6. Understanding of Diabetes in Yi Medicine

Yi nationality is mainly distributed in southwest China. Yi medicine is the result accumulated in the process of adapting to natural conditions. Affected by the local unique living environment, geographical and climatic conditions, and production and living practices, the basic theoretical system of Yi medical understanding of disease is “correspondence between heaven and man” and “Yin and Yang and five elements.” In terms of etiology and pathogenesis, “heat syndrome,” “poison syndrome,” “arrow syndrome,” and “Gu syndrome” were emphasized.

Since there are few records on the understanding of diabetes in Yi medicine and the available information is very limited, it will not be described here.

### 3.2. Preparation of Medicines for the Prevention and Treatment of Diabetes

#### 3.2.1. Analysis on Types of Ethnic Medicinal Resources for the Prevention and Treatment of Diabetes

*Analysis on the Composition of Ethnic Medicine Family and Genus for Prevention and Treatment of Diabetes*. Through the retrieval of relevant books and literature studies, a total of 112 kinds of Tibetan, Mongolian, Miao, Dai, Uygur, and Yi medicines for the prevention and treatment of diabetes were collected and sorted out, involving three categories of plant medicines, animal medicines, and fungal medicines. Among them, 105 kinds of plant medicines come from 52 families, such as Rosaceae, Compositae, Liliaceae, Labiatae, Ranunculaceae, Leguminosae, Cucurbitaceae, Zingiberaceae, Moraceae, Menispermaceae, Amaranthaceae, Berberidaceae, Rutaceae, Gramineae, Campanulaceae, and Orchidaceae; 6 species of animal medicines, from 4 families of Bombycidae, Phasianidae, Apidae, and Cynipidae; and 1 species of fungal medicine, from Polyporaceae. The specific results are shown in Table 1. As can be seen from Table 1, there were seven medicines from Rosaceae. The second is Compositae, Liliaceae, and Lamiaceae, with 6 species, respectively; Ranunculaceae ranks third in number, with 5 species.

**Figure 1:**
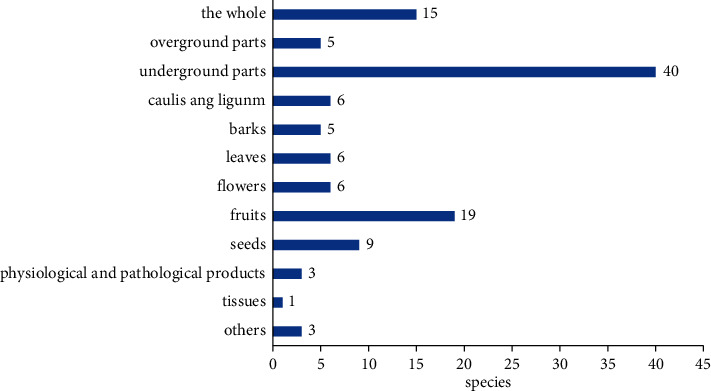
Analysis of medicinal parts of ethnic medicines for the prevention and treatment of diabetes. (Note. When there are multiple medicinal parts of the same medicine, each part is counted separately).

*Analysis of Medicinal Parts of Ethnic Medicines for the Prevention and Treatment of Diabetes.* The medicinal efficacy of medicines is often closely related to their medicinal parts. There may be multiple medicinal parts in the same medicinal material, and different medicinal parts may have different functions. Therefore, the collected 112 kinds of medicinal parts of ethnic medicines used for the prevention and treatment of diabetes were statistically analyzed. The medicinal parts with similar meanings were grouped together, and the frequency of their occurrence was counted. The whole includes the whole plant, the whole grass, and the whole animal; underground parts include plant roots and rhizomes (roots, root tubers, rhizomes, tubers, bulbs); caulis and lignum include the plant vine, stem, and branch; barks include the root bark, tree bark, and branch bark of plants; flowers include plant flowers, style, pollen, and inflorescence; fruits include the fruit, pulp, ear, and peel of the plant; seeds include seeds, kernels, buds, and radicles of plants; physiological and pathological products include physiological and pathological products of animals; and tissues include tissues and organs of animals; in addition, spores, sclerotia, and gall belong to other categories. The specific results are shown in Figure 1.

It can be seen from the figure that the main parts of ethnic medicines for the prevention and treatment of diabetes are underground parts of plant medicines, including roots and underground stems (roots, root tubers, rhizomes, tubers, bulbs), a total of 40 kinds, accounting for 33.90% of the total. Followed by 19 kinds of fruits as medicine, accounting for 16.10% of the total, there were 15 kinds of medicinal materials as a whole, accounting for 12.71% of the total. There were 9 species using seeds as medicine, accounting for 7.63% of the total; there were 6 species of caulis and lignum, and flowers and leaves, accounting for 5.08% of the total. There were 5 species of barks and 5 species of aboveground parts, accounting for 4.24% of the total. There were 3 kinds of physiological products, 3 kinds of pathological products, and 3 kinds of other products, accounting for 2.54% of the total. Animal tissues and organs were only used for 1 drug, accounting for 0.85% of the total.

*Life Type Analysis of Plant Medicines for the Prevention and Treatment of Diabetes.* In the classification of ethnic medicines for the prevention and treatment of diabetes, plant medicines have a large number and occupy a dominant position. In this study, according to the Engler system [33, 34], the plant medicines for the prevention and treatment of diabetes were classified. According to the statistical results, 105 plant drugs were all from 102 species of angiosperms and 3 species of ferns, with a total of 37 orders, 52 families, and 89 genera.

**Figure 2:**
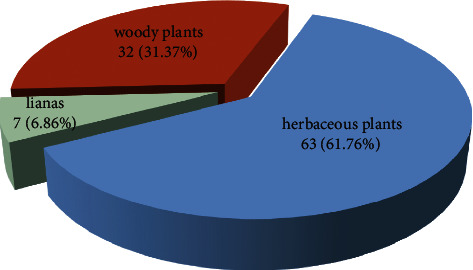
Life type analysis of plant medicines for the prevention and treatment of diabetes.

The medicinal plants were divided into herbaceous plants, lianas, and woody plants, among which one and two annual herbaceous plants, perennial herbaceous plants, and ferns were all classified as herbaceous plants; grassy lianas and woody lianas were all classified as lianas; and shrubs, small trees, and trees were all classified as woody plants [34]. The life forms of 105 medicinal plants used in the treatment of diabetes were statistically analyzed, as shown in Figure 2. According to the statistical results, herbal plants were the main medicinal plants used for the prevention and treatment of diabetes in Tibetan, Mongolian, Miao, Dai, Uygur, and Yi ethnic groups, accounting for 61.76% of the total. The second was woody plants, with 32 species, accounting for 31.37%; there were 7 species of lianas, accounting for 6.86% of the total.

**Figure 3:**
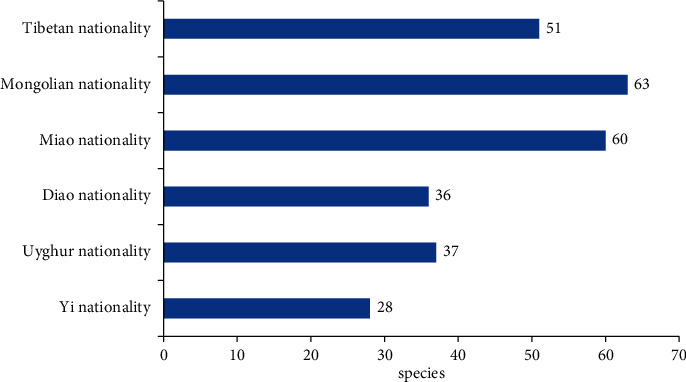
Analysis of the number of medicines for the prevention and treatment of diabetes in different nationalities.

*Analysis of the Number of Medicines for the Prevention and Treatment of Diabetes in Different Nationalities.* According to 112 ethnic medicines for the prevention and treatment of diabetes collected above, there is too much cross-use among different ethnic groups. Therefore, the frequency of their occurrence in various ethnic groups was counted, and the specific results are shown in Figure 3. Among them, there are 51 kinds of drugs for the prevention and treatment of diabetes in Tibetan nationality, mainly Liliaceae, Rosaceae, and Campanulaceae. There are 63 species in Mongolian nationality, mainly Liliaceae. Miao nationality has 60 species, mainly Rosaceae; Dai nationality has 36 species, mainly Zingiberaceae; Uygur nationality has 37 species, mainly Liliaceae; and Yi nationality has 28 species, mainly Rosaceae. Most of the medicinal parts of medicinal materials used by the abovementioned ethnic groups came from underground parts of plants.

#### 3.2.2. Ethnodrug for Diabetes in Modern Research

Due to the excessive number of ethnic medicines for the prevention and treatment of diabetes, the whole list is too long. Therefore, based on the keywords of “medicinal materials name,” “diabetes,” and “antihyperglycemic,” 30 kinds of medicinal materials with many literature studies and reports were retrieved and summarized, and the active ingredients related to the treatment of diabetes and its complications were described according to the current literature studies and reports. The specific results are shown in Table 2. It listed in detail 3 kinds of medicinal materials, which are most studied in modern times. As can be seen from Table 2, the active ingredients related to the treatment of diabetes mainly include polysaccharide, saponins, terpenoids, flavonoids, alkaloids, and other chemical structure types. Most of the active ingredients are not single chemical components, but a group of active synergistic ingredients.

*Astragalus.* This product is the dried root of Astragalus membranaceus (Fisch.) Bge. var. mongholicus (Bge.) Hsiao or Astragalus membranaceus (Fisch.) Bge. It is widely used in Tibetan and Mongolian areas.

The main chemical components of Astragalus membranaceus are polysaccharides, saponins, flavonoids, amino acids, trace elements, sterols, and so on. Polysaccharides, saponins, and flavonoids all have hypoglycemic effect. Zhang et al. [3] found that polysaccharide may improve insulin sensitivity by activating AMPK in 3T3-L1 adipocytes, thereby enhancing glucose uptake. Xue et al. [35] proved that polysaccharide can significantly reduce blood glucose and protect renal function in the diabetic rat model. Lin et al. [36] found that astragaloside IV can reduce the levels of blood glucose, triglyceride, and insulin in type 2 diabetic mice and inhibit the mRNA and protein expression and enzyme activity of liver glycogen phosphorylase (GP) and glucose 6-phosphatase (G6Pase). In recent years, the popular etiology theory of diabetes inflammation believes that diabetes is a kind of natural immune and low-grade inflammatory disease, and cytokine-mediated inflammatory response affects insulin secretion by inducing apoptosis and dysfunction of pancreatic *β*-cells [37]. Zhu et al. [38] found that TFA of Astragalus membranaceus can reduce the inflammation level of HK-2 cells induced by high glucose.

To sum up, there are many literature studies and reports proving that polysaccharides, saponins, and flavonoids contained in Astragalus membranaceus have hypoglycemic effects. These components can improve the expression of damaged cells and genes in high glucose state, regulate oxidative stress and anti-inflammatory effects, improve vascular endothelial function, regulate metabolism, and so on, to provide symptomatic regulation for diabetes and its complications.

*Puerarin* Dried Root of Leguminous Plant *Pueraria lobata* (Willd.) Ohwi. It has the reputation of “Asian ginseng.” It is mainly used by Mongolians.

There are many chemical components in Pueraria root, mainly including isoflavones, triterpenoids, saponins, and polysaccharides [39]. Puerarin was the main isoflavone in Pueraria root. Wang et al. [40] significantly improved glucose homeostasis and observed markers of new *β*-cell formation (insulin, PDX1, and Ngn3) in the pancreatic ducts of high-fat diet-induced diabetic mice treated with Puerarin, suggesting that Puerarin can promote *β*-cell regeneration through GLP-1R signal activation and improve hyperglycemia in HFD diabetic mice. Xu et al. [41] established a rat model of gestational diabetes by high-fat diet combined with intraperitoneal injection of streptozotocin and found that Puerarin exerts anti-inflammatory effects by downregulating TLR4/MyD88/NF-*κ*B inflammatory signaling pathway in gestational diabetic rats. Zhang et al. [42] found that high, medium, and low doses of Puerarin could significantly reduce the blood glucose level and the incidence of cataract in diabetic rats by intraperitoneal injection. In addition, Puerarin was found to inhibit oxidative stress and restore malondialdehyde and glutathione levels and glutathione peroxidase activity. Chen [43] also pointed out that Puerarin can reduce blood glucose level, improve insulin resistance, protect islets, inhibit inflammation, reduce oxidative stress, and inhibit the formation of Maillard reaction and advanced glycation end products (AGEs) and may also delay and improve a series of complications of diabetes.

In conclusion, Pueraria root may be a potential adjunctive drug for treating diabetes and diabetic complications in the future.

*Coptis* Dried Roots of *Coptis chinensis* Franch., *Coptis deltoidea* C. Y. Cheng et Hsiao, or *Coptis teeta* Wall. It is widely used in Tibetan, Mongolian, Uygur, and other areas.

At present, more than 100 kinds of chemical components have been isolated from Coptis chinensis, including alkaloids, lignans, flavonoids, and acidic components [44]. Coptisine and berberine are the main hypoglycemic agents in Coptis chinensis, while parmatine, jatrorrhizine, and epiberberine play different synergistic roles [45]. Berberine is the most important and representative hypoglycemic component with a content of 5%–8% and has been clinically proven to be safe and effective in the treatment of type 2 diabetes [46]. Modern studies have shown that berberine can reduce blood glucose in type 2 diabetic patients by increasing insulin receptor expression and increasing glucose efficacy [47] or alleviate hyperglycemia by inhibiting the hepatic glucagon pathway in diabetic mice [48]. It has also been found that berberine can improve diabetic inflammation in ZDF rats and insulin-resistant HepG2 cells through the PPM1B pathway [49]. Therefore, the mechanism of berberine in the treatment of diabetes is not unique, and it is through the synergistic action of multiple targets. In addition, berberine can also achieve the purpose of treating diabetes by regulating intestinal microflora [50], regulating lipid metabolism [51], and inhibiting liver glycogenosis [52] and oxidative stress [53].

## 4. Deficiencies and Prospects

Ethnic medicines are mainly derived from natural plants, animals, or minerals. Compared with chemical drugs, their toxic and side effects are much smaller. Most ethnic drugs are non-toxic or low toxicity, and a few are toxic or extremely toxic, but it is definitely not the so-called general “ethnic drugs are natural drugs, without any toxic and side effects.” For example, the main components of rhubarb in reducing blood sugar are rhein and emodin and other anthraquinones. However, modern studies have shown that anthraquinones contained in rhubarb are toxic to the liver and kidney. Ren et al. [54] administered 10 g/(kg·d) self-made rhubarb total anthraquinone (2.28%) by intragastric administration to normal rats and found that renal insufficiency, severe edema and necrosis of renal tubular epithelial cells, and renal damage were induced in the high-dose group. WU et al. [55] studied gender differences in emodin hepatotoxicity and toxicokinetics and concluded that emodin can cause hepatotoxicity in rats, and liver damage in female rats is more obvious than that in male rats. Although the ethnic medicine for the prevention and treatment of diabetes is not absolutely safe, it is not reasonable to talk about poison and alarmist, and it is unreasonable to discuss the toxicity of any drug without dosage. As long as under the guidance of the principle of syndrome differentiation, the incompatibility of drugs should be paid attention to, and the dosage and course of medication should be rationally determined, the good therapeutic effect can be achieved, and the toxic and side effects can be avoided or reduced.

Ethnic medicine is the sum of medical theories, technical skills, and materials gradually formed and developed by a nation in its living environment and long-term struggle against diseases, which is extremely regional. It is an important part of the world medicine. Treasury has made important contributions to the health of the people in ethnic areas. The medical system and drugs for treating diabetes of Tibetan, Mongolian, Miao, Dai, Uygur, and Yi nationalities are only reviewed in this study, and more nationalities are not involved. At present, systematic research reports on the pharmaceutical theory and virus theory of diabetes in most ethnic groups are not perfect. Due to historical factors such as ethnic writing and language, some ancient books of ethnic medicine have been damaged or lost. With the passage of time, many predecessors left the special effects of ethnic medicine, and experience and folk remedies are gradually lost and cannot be verified. At present, the effective components of many ethnic drugs are unknown and lack modern scientific research, which affects the formulation of drug quality standards and the development of new drugs. For example, Zhou et al. [56] pointed out that there were many successful cases of diabetic foot infection treated with rape oil after porcupine quills were burned and ground into powder in Miao people's living areas. However, there is little research on the chemical composition of this drug, only limited to protein, amino acid, and nutritional analysis. There are no reports on the characteristic pharmacodynamic components, content determination, and basic research of pharmacodynamic substances, which to some extent restrict the development and clinical use of its preparations. Therefore, it is urgent to increase and speed up the special investigation and directional protection of traditional knowledge of ethnic medicine, timely and systematically excavate, sort out, and translate ethnic medical classics and books, and strengthen the theoretical, basic, and clinical research of ethnic medicine. It is also of great significance to enrich the connotation of ethnic medicine and explore and develop candidate drugs for treating modern incurable diseases.

The understanding of diabetes among Tibet, Mongolia, Miao, Dai, and Uygur is “almost the same.” Although the ethnic terms are different, they all believe that diabetes is caused by internal and external factors of the body, and the essence cannot be transformed due to the dysfunction of viscera and excreted with urine. This may be because of the frequent migration and integration of ethnic groups in history, the frequent interaction and communication of medicine, and mutual learning from each other's strengths, so there is “great harmony” in the medicine of all ethnic groups. In addition, the distribution of ethnic groups is characterized by large dispersion and small settlement, so there are “small differences” in the medical theories of ethnic groups and even in the medication experience of different regional groups. Thus, it appears that each nationality retains its own ethnic characteristics while having similar medical theories.

To sum up, ethnic medicine is not stuck in its own ways, but a discipline is developed by absorbing the essence of Han medicine and other contents, integrating its own environment and culture, and gathering the wisdom of predecessors. Ethnic medicine has abundant resources in diabetes treatment and has excellent development prospects, which is worthy of further exploration and modern research.

## Figures and Tables

**Table 1:** Analysis on the composition of ethnic medicine family and genus for prevention and treatment.

Family name	Amount	Family name	Amount	Family name	Amount
Rosaceae	7	Myrtaceae	2	Nymphaeaceae	1
Compositae	6	Araliaceae	2	Araceae	1
Liliaceae	6	Phasianidae	2	Celastraceae	1
Labiatae	6	Bombycidae	2	Cactaceae	1
Ranunculaceae	5	Combretaceae	1	Scrophulariaceae	1
Leguminosae	4	Ebenaceae	1	Convolvulaceae	1
Cucurbitaceae	4	Eucommiaceae	1	Urticaceae	1
Zingiberaceae	4	Juglandaceae	1	Iridaceae	1
Moraceae	4	Crassulaceae	1	Myrsinaceae	1
Menispermaceae	3	Chenopodiaceae	1	Nyctaginaceae	1
Amaranthaceae	3	Portulacaceae	1	Arecaceae	1
Berberidaceae	3	Loganiaceae	1	Apiaceae	1
Rutaceae	3	Geraniaceae	1	Lygodiaceae	1
Gramineae	2	Rubiaceae	1	Polypodiaceae	1
Campanulaceae	2	Solanaceae	1	Adiantaceae	1
Orchidaceae	2	Cornaceae	1	Polyporaceae	1
Polygonaceae	2	Amaryllidaceae	1	Musaceae	1
Cruciferae	2	Plantaginaceae	1	Apidae	1
Punicaceae	2	Euphorbiaceae	1	Cynipidae	1

**Table 2:** Ethnodrug for diabetes in modern research (the order of medicine names is from high to low according to the number of studies reported).

No	Chinese name	Latin name of the original plant	Family	The nationality that uses it	Medicinal part	Bioactive components related to diabetes mellitus were reported
1	Huang Qi	*Astragalus membranaceus* (Fisch.) Bge.	Leguminosae	Tibetan, Mongolian	Root	Polysaccharide, saponin, flavone
2	Ge Gen	*Pueraria lobata* (Willd.) Ohwi	Leguminosae	Mongolian	Root	Puerarin
3	Huang Lian	*Coptis chinensis* Franch.	Ranunculaceae	Tibetan, Mongolian, Uygur	Rhizome	Berberine, polysaccharide, coptisine
4	Di Huang	*Rehmannia glutinosa* Libosch.	Scrophulariaceae	Mongolian, Dai	Earthnut	Catalpol, polysaccharide
5	Yu Zhu	*Polygonatum odoratum* (Mill.) Druce	Liliaceae	Tibetan, Mongolian, Uygur	Rhizome	Polysaccharide, flavone
6	Sang Ye	*Morus alba* L.	Moraceae	Miao, Dai	Leaf	Polysaccharide, flavone, alkaloid
7	Da Huang	*Rheum officinale* Baill.	Polygonaceae	Tibetan, Mongolian, Uygur	Root and rhizome	Emodin, rhein
8	Nan Gua	*Cucurbita moschata* Duchesne	Cucurbitaceae	Yi, Dai	Pulp	Polysaccharide
9	Gou Qi	*Lycium chinense* Mill.	Solanaceae	Tibetan, Mongolian, Uygur, Miao	Fruit	Polysaccharide
10	Hong Hua	*Carthamus tinctorius* L.	Composite	Tibetan, Mongolian, Uygur, Dai	Flower	Safflower yellow
11	Jiang Huang	*Curcuma longa* L.	Zingiberaceae	Tibetan, Uygur, Miao, Dai	Rhizome	Curcumin
12	Jiao Gu Lan	*Gynostemma pentaphyllum* (Thunb.) Makino	Cucurbitaceae	Miao, Dai	Overground part	Saponin
13	Hong Jing Tian	*Rhodiola rosea* Linn.	Crassulaceae	Tibetan	Root and rhizome	Salidroside
14	Luo Han Guo	*Siraitia grosvenorii* (Swingle) C. Jeffrey ex A. M. Lu et Z. Y. Zhang	Cucurbitaceae	Miao	Fruit	Saponin, polysaccharide
15	Zhi Mu	*Anemarrhena asphodeloides* Bge.	Liliaceae	Mongolian	Rhizome	Mangiferin
16	Huang Qin	*Scutellaria baicalensis* Georgi	Lamiaceae	Mongolian, Miao	Root	Baicalin, wogonin
17	Bai Zhu	*Atractylodes macrocephala* Koidz.	Composite	Miao	Rhizome	Volatile oil, lactone
18	Ma Chi Xian	*Portulaca oleracea* L.	Portulacaceae	Tibetan, Uygur, Miao, Dai, Yi	Overground part	Polysaccharide
19	Gan Cao	*Glycyrrhiza uralensis* Fisch.	Leguminosae	Tibetan, Mongolian, Uygur, Dai	Root and rhizome	Glycyrrhizic acid, flavone
20	Mai Dong	*Ophiopogon japonicus* (L. f) Ker Gawl.	Liliaceae	Mongolian, Uygur, Miao	Earthnut	Polysaccharide
21	Shan Zhuyu	*Cornus officinalis* Sieb. et Zucc.	Cornaceae	Mongolian, Miao	Pulp	Total iridoid glycosides of Cornus officinalis
22	Ku Qiao Mai	*Fagopyrum tataricum* (L.) Gaertn.	Polygonaceae	Dai, Miao, Yi, Tibetan	Whole grass or root	Flavone
23	Yi Yi Ren	*Coix lacryma-jobi* L.	Gramineae	Tibetan, Mongolian	Seed kernel	Coix seed polysaccharides A, B, C
24	Xian Ren Zhang	*Opuntia dillenii* (Ker Gawl.) Haw.	Cactaceae	Miao, Dai, Yi	Fleshy stem	Polysaccharide
25	Jin Ying Zi	*Rosa laevigata* Michx.	Rosaceae	Mongolian, Miao	Fruit	Saponin
26	Fu Ling	*Poria cocos* (Schw.) Wolf	Polyporaceae	Tibetan, Mongolian, Miao	Sclerotium	Polysaccharide
27	Shi Ye	*Diospyros kaki* Thunb.	Ebenaceae	Miao	Leaf	Flavone
28	Du Zhong	*Eucommia ulmoides* Oliv.	Eucommiaceae	Mongolian, Miao	Bark	Polysaccharide, flavone
29	Hu Lu Ba	*Trigonella foenum-graecum* L.	Leguminosae	Tibetan, Mongolian, Uygur	Seed	Fenugreen
30	Huang Bo	*Phellodendron chinense* Schneid.	Rutaceae	Mongolian, Miao	Bark	Berberine

## References

[1] Williams R., Karuranga S., Malanda B. (2020). Global and Regional Estimates and Projections of Diabetes-Related Health Expenditure: Results from the International Diabetes Federation Diabetes Atlas. *Diabetes Research and Clinical Practice*.

[2] Yang H. F. (2015). Understanding and treatment of diabetes mellitus in Tibetan medicine. *Diabetes New World*.

[3] Zhang R., Qin X., Zhang T. (2018). Astragalus polysaccharide improves insulin sensitivity via AMPK activation in 3T3-L1 adipocytes. *Molecules*.

[4] Chen X.-F., Li X. L., Yang M., Song Y., Zhang Y. (2018). Osteoprotective effects of salidroside in ovariectomized mice and diabetic mice. *European Journal of Pharmacology*.

[5] Yuan R. Y., Zhuoma D. Z., Zeren L. M. (2021). Research progress of Tibetan medicine on the therapeutic effect of diabetes mellitus and its complications. *Plateau scientific research*.

[6] Wang Y. R., Zhao C. H., Wangman J. C. (2013). Treatment of diabetes mellitus by Tibetan medicine. *Chinese Journal of Ethnic Medicine*.

[7] Gao J., Pan L., Bi R. H. (2021). Tibetan medicines and Tibetan prescriptions for the treatment of diabetes mellitus. *Evidence-based Complementary and Alternative Medicine*.

[8] Pa K. (1989). *Eight Branches Essentials*.

[9] Zhong J. G. (2014). Tibetan medicine’s understanding and treatment of type 2 diabetes. *Tibetan studies in China*.

[10] Yang H. F. (2015). Tibetan medicine’s understanding and treatment of diabetes. *New world of diabetes*.

[11] Ethnic Medicine Research Group of Central University for Nationalities (2013). *Ethnic Medicine*.

[12] Li K. (2007). *Four Necter Treatises of Mongolian Medicine*.

[13] Wuliji A. (1989). *BZ Mongolian Medicine Internal Medicine*.

[14] Qiqi G. T. (1983). *Selected Mongolian Medicine*.

[15] Bai J. Q. (2019). Brief introduction of Mongolian medicine’s understanding of diabetes. *Chinese Journal of Ethnic Medicine*.

[16] Yong H., Ni H. (2019). Understanding of diabetes in Mongolian medicine. *World Latest Medical Information Abstract*.

[17] Jakhadai C. (2012). *Encyclopedia of Mongolian Studies·Medicine*.

[18] Saren G. W., Bao H. J. (2020). Research progress of Mongolian medicine in the treatment of diabetes. *Chinese Journal of Ethnic Medicine*.

[19] Du J., Zhang J. M. (2007). *Miao Medical Foundation*.

[20] Fu J., Cui J., Guo W. W. (2017). Analysis of “challenge therapy” of miao doctor’s “poison” Theory. *Chinese Journal of Ethnomedicine and Ethnopharmacy*.

[21] Li D., Chen Y. H., Xu S. H. (2020). A brief analysis of the application of “poison” theory in Miao Medicine in quenching thirst and removing gangrene. *Hunan Journal of Traditional Chinese Medicine*.

[22] Deng M. X., Chen Y. H., Ni H. G. (2021). Application of miao medicine, Posion “Theroy in Diabetes”. *Asion-Pacific Traditional Medicine*.

[23] Fu L. H., Zhou X., Ge Z. X. (2019). Investigation and study on the drugs commonly used in the internal treatment of miao Medicine. *Chinese Journal of Ethnomedicine and Ethnopharmacy*.

[24] Chen J., Yang H. (2016). Clinical experience of Tangningtongluo capsule in the treatment of diabetic foot. *China Medical Review*.

[25] Zhang C. (2007). *Basic Theory of Dai Medicine*.

[26] Niu F., Zhang C., Zheng J. (2009). Discussionon the relationship between the theory of four elements and five skandhas and the theoryof Dai medicione disease. *Journal of Yunnan University of Traditional Chinese Medicine*.

[27] Zhang Z., Tian C. H. (1992). Comparative study of Dai medicine theory. *Yunnan Journal of Traditional Chinese Medicine*.

[28] Li J., Lin L. (2021). Research status of Dai Medicine in the treatment of endocrine and metabolic diseases. *China’s Naturopathy*.

[29] Tang Y. X. (1992). On the relationship between the theory of four elements and five skandhas of Dai medicine and human diseases. *Yunnan Journal of Traditional Chinese Medicine*.

[30] Zhang Z., Tian C. H. (1992). A comparative study of Dai medicine theory (continuation 1). *Yunnan Journal of Traditional Chinese Medicine*.

[31] Zhu H. M. (2008). Clinical progress of ethnic medicine in treating diabetes. *Chinese Journal of Ethnomedicine and Ethnopharmacy*.

[32] Guan S., Chen L., Guo X. Y. (2014). Treatment of diabetes with Uygur medicine. *China’s Naturopathy*.

[33] Abuduwaili A. B. (2009). Uyghur Medical Guide for diagnosis and treatment of diabetes. *Chinese Journal of Ethnomedicine and Ethnopharmacy*.

[34] Zhou Y. (2016). *Investigation on traditional medicinal plants for prevention and treatment of diabetes in China*.

[35] Xue M., Wei M. M., Wang D. (2020). Astragalus polysaccharides protect renal function and affect the TGF-*β*/Smad signaling pathway in streptozotocin-induced diabetic rats. *Journal of International Medical Research*.

[36] Lin L., Wu S. Y., Wang G. F. (2010). Effect of astragaloside IV on hepatic glucose-regulating enzymes in diabetic mice induced by a high-fat diet and streptozotocin. *Phytotherapy Research: PTR*.

[37] Wei Q. F., Wang H. X. (2020). Research Progress in astragalus Total Flavone Multi-Target Regulation for Diabetes mellitus and its complications. *Central South Pharmacy*.

[38] Zhu G. H., Wang X. W., Qi C. (2019). The regulatory effect of Astragalus total flavonoids on high glucose induced inflammatory factors in HK-2 cells through miR-378. *Immunological Journal*.

[39] Li S. X. (2020). Research progress of chemical constituents and pharmacological effects of pueraria lobata. *Liaoning Chemical Industry*.

[40] Wang C. J., Yao J. H., Ju L. J. (2020). Puerarin ameliorates hyperglycemia in HFD diabetic mice by promoting *β*-cell neogenesis via GLP-1R signaling activation. *Phytomedicine: International Journal of Phytotherapy and Phytopharmacology*.

[41] Xu W., Tang M., Wang J., Wang L. (2020). Anti-inflammatory activities of puerarin in high-fat diet-fed rats with streptozotocin-induced gestational diabetes mellitus. *Molecular Biology Reports*.

[42] Zhang D. Z., Li M. (2019). Puerarin prevents cataract development and progression in diabetic rats through Nrf2/HO-1 signaling. *Molecular Medicine Reports*.

[43] Chen X., Yu J., Shi J. (2018). Management of diabetes mellitus with puerarin, a natural isoflavone from pueraria lobata. *The American journal of Chinese medicine*.

[44] Lin F., Qiang F., Ji L. (2021). Research progress on chemical Constituents and pharmacological action of Coptis chinensis. *Acta Chinese Medicine and Pharmacology*.

[45] Ma H., Hu Y. R., Zou Z. Y. (2019). Preliminary evaluation of antihyperglycemic effect of Rhizoma Coptidis alkaloids and their structure-activity relationships. *Chinese Pharmacological Bulletin*.

[46] Yin J., Xing H., Ye J. (2008). Efficacy of berberine in patients with type 2 diabetes mellitus. *Metabolism*.

[47] Zhang H., Wei J., Wu R. (2010). Berberine lowers blood glucose in type 2 diabetes mellitus patients through increasing insulin receptor expression. *Metabolism*.

[48] Zhong Y., Jin J., Liu P. Y. (2020). Berberine attenuates hyperglycemia by inhibiting the hepatic glucagon pathway in diabetic mice. *Oxidative Medicine and Cellular Longevity*.

[49] Wu Y. S., Li Z. M., Chen Y. T. (2020). Berberine improves inflammatory responses of diabetes mellitus in zucker diabetic fatty rats and insulin-resistant HepG2 cells through the PPM1B pathway. *Journal of immunology research*.

[50] Zhang X., Zhao Y., Zhang M. (2012). Structural changes of gut microbiota during berberine-mediated prevention of obesity and insulin resistance in high-fat diet-fed rats. *PLoS One*.

[51] Kong W., Wei J., Abidi P. (2004). Berberine is a novel cholesterol-lowering drug working through a unique mechanism distinct from statins. *Nature Medicine*.

[52] Jiang S.-J., Dong H., Li J. B. (2015). Berberine inhibits hepatic gluconeogenesisviathe LKB1-AMPK-TORC2 signaling pathway in streptozotocin-induced diabetic rats. *World Journal of Gastroenterology*.

[53] Zhou J., Zhou S., Tang J. (2009). Protective effect of berberine on beta cells in streptozotocin- and high-carbohydrate/high-fat diet-induced diabetic rats. *European Journal of Pharmacology*.

[54] Ren H. B., Wang Y. Y., Wang Tuanjie T. J. (2012). Study on acute renal toxicity of rhubarb total anthraquinone in rats. *Journal of liaoning university of traditional Chinese medicine*.

[55] Wu L., Han W., Chen Y. (2018). Gender differences in the hepatotoxicity and toxicokinetics of emodin: The Potential Mechanisms Mediated by UGT2B7 and MRP2. *Molecular Pharmaceutics*.

[56] Zhou B. H., Tang J. P., Li X. R. (2020). Research progress on comprehensive utilization of animal medicine porcupine quill. *Asia-pacific traditional medicine*.

